# The 9th annual INDUS-EM 2013 Emergency Medicine Summit, “Principles, Practices, and Patients,” a level one international meeting, Kerala University of Health Sciences and Jubilee Mission Medical College and Research Institute, Thrissur, Kerala, India, October 23–27, 2013

**DOI:** 10.1186/1747-5341-9-8

**Published:** 2014-05-06

**Authors:** Mamta Swaroop, Sagar C Galwankar, Stanislaw PA Stawicki, Jayaraj M Balakrishnan, Tamara Worlton, Ravi S Tripathi, David P Bahner, Sanjeev Bhoi, Colin Kaide, Thomas J Papadimos

**Affiliations:** 1 Northwestern University Feinberg School of Medicine, 676 North Saint Clair Street, Chicago, IL 60611, USA; 2 Faculty of Medicine and Global Health, University of South Florida, 12901 Bruce B. Downs Blvd, Tampa, FL, USA; 3 Department of Surgery, The Ohio State University Wexner Medical Center, 410 West 10th Avenue, Columbus, OH 43210, USA; 4 Department of Emergency Medicine, Jubilee Mission Medical College and Research Institute, Thrissur, Kerala, India; 5 Department of Surgery, Walter Reed National Military Medical Center, 8901 Wisconsin Avenue, Bethesda, MD 20889, USA; 6 Department of Anesthesiology, The Ohio State University Wexner Medical Center, 410 West 10th Avenue, Columbus, Ohio 43210, USA; 7 Department of Emergency Medicine, The Ohio State University Wexner Medical Center, 410 West 10th Avenue, Columbus, Ohio 43210, USA; 8 Department of Emergency Medicine, JPN Apex Trauma Centre, All India Institute of Medical Sciences, New Delhi 110 029, India

**Keywords:** Medical education, Emergency medicine, Public health, Philosophy medical, World health

## Abstract

INDUS-EM is India’s only level one conference imparting and exchanging quality knowledge in acute care. Specifically, in general and specialized emergency care and training in trauma, burns, cardiac, stroke, environmental and disaster medicine. It provides a series of exchanges regarding academic development and implementation of training tools related to developing future academic faculty and residents in Emergency Medicine in India. The INDUS-EM leadership and board of directors invited scholars from multiple institutions to participate in this advanced educational symposium that was held in Thrissur, Kerala in October 2013.

## Introduction

From October 23 to October 27, 2013 the 9th annual INDUS-EM conference entitled “Principles, Practices, and Patients,” was held in Thrissur, Kerala, India. The conference faculty was composed of physicians from India, the United States of America, Israel, and the United Kingdom. The conference had over 2,800 attendees and was aimed towards medical students, nursing students, residents, and junior faculty. The focus of the international faculty and participants at this conference was two-fold; The first portion of this effort was (1a) to provide a two day pre-conference research symposium (EM-LEADER--emergency medicine leadership in education and academic development via enterprising research), whereby students, residents and junior faculty would be introduced to concepts of how to conduct successful research through the acquisition of research funding by successful grant writing, and how to successfully navigate administrative quagmires, and (1b) to understand and be aware of the basic concepts of statistics, epidemiological methods, use of the electronic medical record, how to write a manuscript, and other scientific methods through specific examples, while at the same time attempting to imbue an ethical thread throughout the scientific curriculum; especially in regard to the protection of human subjects participating in clinical trials and studies, and the protection of their personal information. The second portion of international participation in regard to education and consultation was a trauma and critical care workshop where techniques of airway access, venous and arterial access, methods of monitoring, diagnostic methods, and clinical evaluation of the critically ill patient were highlighted. This curriculum was presented during the Summit Program (see Table 1 for faculty).

**Table 1:** Faculty participants

**EM-LEADER**^**a**^	**FORT**^**b**^	**TRCCRC**^**c**^
Arquilla, B^5^	Anderson, HL III^10^	Anderson, HL III^10^
Bahner DP^2, 3^	Kaide, CG^3^	Bahner, DP^2, 3^
Bhoi, S^6^	Kataria, H^8^	Kaide, CG^3^
Galwankar, SC^12^	Kumar, V^7^	Michaelson, M^13^
Paladino, M^5^	Manohar, R^9^	Dev Soni, K^6^
Papadimos, TJ^2, 3^	Michaelson, M^13^	Stawicki, SPA^2, 3^
Papanagou, D^5^	Stawicki, SPA^2, 3^	Swaroop, M^1, 2^
Pohlman, K^5^	Swaroop, M^1, 2^	Tripathi, RS^3^
Sanson, T^4^	Worlton, T^2, 11^	Worlton, T^2, 11^
Stawicki, SPA^2, 3^		
Swaroop, M^1, 2^		

^a^Emergency Medicine Leadership in Education and Academic Development via Enterprising Research, ^b^Fundamentals of Trauma Resuscitation, ^c^Trauma Critical Care Resuscitation Course. Academic affiliations: ^1^Northwestern University, Chicago, IL, ^2^OPUS 12 Foundation, Inc.; ^3^Ohio State University Wexner Medical Center, Columbus, OH; ^4^University of South Florida, Tampa, FL; ^5^State University of New York (SUNY)-Downstate, Brooklyn, NY; ^6^All India Institute of Medical Sciences, New Dehli, India; ^7^Lokmanya Tilak Municipal Medical College and General Hospital, Sion, Mumbai, India; ^8^St. Helen’s and Knowsley Teaching Hospitals NHS Trust; ^9^Wirral University Hospitals NHS Trust, Northwest UK; ^10^St. Joseph Mercy Medical Center, Ann Arbor, MI; ^11^Uniformed Services University of the Health Sciences, Bethesda, MD; ^12^Consultant for Emergency Medicine, Global Hospitals, India, Assistant Professor, University of South FL and Indus-Em 2013 Conference Director; ^13^Rambam Health Care Campus, Haifa, Israel.

Since 2008 the INDO-US joint working group (JWG) has made progress regarding emergency medicine (EM) (1). Medicine in India, and particularly EM, still faces an acute self-imposed ethical-societal challenge. While there have been improvements recently, India lacks an emergency service system that is responsive, effective, and time sensitive [1]. It lacks a countrywide uniform emergency/ambulance number (forthcoming…dial 108). Nearly 30% of emergency patients in India die before reaching hospital and 80% of trauma victims are not cared for within the first post-injury *golden hour*[2]; only 2% of patients were transported to the Emergency Department (ED) by ambulance [3]. Since there are few trauma providers in India and few trauma centers, most emergency care in India occurs in the *casualty* or *accident* rooms where specialty training and formal education in EM, trauma, and critical care often do not exist [1]. Furthermore, India is a land of frequent natural and human catastrophes: floods, cyclones, tsunamis, earthquakes, droughts, and terrorism. Pre-hospital care is rare and inadequate. One survey reported that only 26% of the health systems have a well-prepared disaster plan [4].

The INDO-US Joint Working Group (JWG) and like-minded, Indian-allied physicians have embarked on an educational journey through the vehicle of an annual INDUS-EM educational summit. With the hopes of reaching the hearts and minds of the medical and nursing students, residents, other young medical professionals, and faculty members, they intended to change the philosophy of the approach of the Indian medical community to modern EM. Furthermore, they hoped to make an ethical precedent to provide appropriate and outstanding care to India’s citizens and visitors by formally trained specialists in well-equipped facilities. This report of the 9th INDUS-EM LEADER conference summarizes the educational efforts made toward the above-mentioned philosophy and ethical precept.

The meeting was broken into two specific portions, (1) a pre-summit meeting entitled, EM-LEADER (emergency medicine leadership in education and academic development via enterprising research), which covered academic leadership skills, clinical research, publication, the electronic medical record, and grant writing; and (2) the main summit.

### First day of EM-LEADER

Dr. Mamta Swaroop, Assistant Professor of Surgery in Trauma and Critical Care from Northwestern University, Chicago, Illinois, USA and the Co-Chair of the OPUS 12 Foundation’s International Medicine Expert Group started the first day of the pre-summit research conference with a researcher’s perspective of the skills needed to publish in an academic medical center, focusing on leadership and mentorship aspects of the academia. She additionally lectured on the topics of patient and data confidentiality and their vital importance in conducting ethical research.

Dr. Stanislaw P.A. Stawicki, Associate Professor of Surgery and Director of Research in the Division of Trauma, Critical Care, and Burn at The Ohio State University, Columbus, USA presented several lectures. His first lecture revolved around the need to pursue funding of research through sources in industry to alleviate the increasing lack of resources from local, state, and federal government. He advised the audience on how to pursue such finding, and the importance of the ethical standards that were needed when collaborating with the private sector in regard to presentation and publication of results. His second lecture was an examination of the necessary approach required to receive approval for research in academic medical centers from an organization’s Institutional Review Board (IRB) and from Ethics boards. The third lecture explained how to organize an original research manuscript. His final lecture of the day covered plagiarism and academic dishonesty and its implications regarding both individuals and institutions.

Dr. David P. Bahner, Professor of EM from The Ohio State University, Columbus, OH, USA gave a perspective on the logistics of the use of office staff and trainees in pursuing research and education, especially in regard to their value and appreciation of collaborative efforts to pursue academic research and excellence. Dr. Bahner’s second lecture was a discourse on ethical authorship sharing and its importance to collaborative research efforts. His third lecture involved an explanation of how to write a review article.

Dr. Thomas J. Papadimos, Professor of Anesthesiology, Vice Chair for Academic Affairs, Section Director of Anesthesiology Critical Care Medicine, and Director of Critical Care Service at the Ross Heart Hospital at The Ohio State University, Columbus, OH, USA started his lecture block with an important discussion regarding clinical colleagues and a faculty member’s relationship to the institution’s administration. His second lecture was on the topic of intellectual property; what it is, how it affects the academician-inventor, and to what to expect and not to expect from an institution’s office of technology. His third lecture involved the techniques of data collection and storage, the associated pitfalls, and the associated important and necessary ethical considerations. His final presentation of day one of the pre-summit explained how to write a letter to the editor and how to write case reports.

Dr. Lorenzo Paladino, Assistant Director of Research at the State University of New York (SUNY)-Downstate, Brooklyn, New York, USA presented a lecture on researching overcrowding, violence, and abuse; its methods, and ethics. He provided a second lecture on the basics of data analysis regarding tests and software.

Dr. Dimitrios Papanagnou, Assistant Professor of EM and the State University of New York (SUNY), Brooklyn, New York, USA and Mr. Kevin Pohlman, CHSE, NREMT-P, a simulation specialist, presented an informative session on the evaluation of educational tools to improvise teaching, especially the ultrasound use.

### Second day of EM -LEADER

The first lecture was presented by Dr. Sanjeev Bhoi of the All India Institute of Medical Sciences (AIIMS), New Dehli, India. His presentation on the use of electronic medical records in the process of development and research in EM and Trauma was well received. He further delivered an update on the progress of the electronic medical record in India.

Dr. Sagar C. Galwankar, Chief of EM, Global Hospitals, India, Assistant Professor, University of South Florida, Tampa and the Scientific Chairman of the INDUS-EM 2013 Conference, then gave a detailed lecture on ethical animal research and how the US medical system handles animals in its biomedical investigative processes. Dr. Stawicki then began the grantsmanship seminar with a lecture entitled *Asking the Right Research Question*. He followed later in the afternoon with a lecture on patient safety and how to design and conduct patient safety research. Dr. Papadimos then gave an extensive lecture on grantsmanship which included the idea/hypothesis process, searching for funding, content of a proposal, writing skills, alliances to strengthen the application, budget and justification, support letters, and building a resume. This was followed by a panel discussion with Dr. Papadimos, Dr. Stawicki, Dr. Bhoi, and Dr. Galwankar.

Dr. Bonnie Arquilla, Associate Professor of EM, State University of New York (SUNY), Brooklyn, New York gave a session focusing on mass casualty situations research. Dr. Arquilla was followed by Dr. Tracy Sanson, Associate Professor of EM and Emergency Residency Program Director at the University of South Florida, Tampa, Florida, who gave a lecture on presentation and communications skills.

The EM-LEADER session ended with a round table discussion of research ideas for the Indian network of Critical Illness and Injury Translational Trial Experts (INCIITTE) led by Dr. Sagar Galwankar and Dr. Sanjeev Bhoi of AIIMS, New Delhi, India.

### The summit program

The summit program was expansive and varied regarding topics of discussion. Due to the limited scope of this article, the reader is referred to the INDUS-EM 2013 Internet Website for further details regarding the Summit [5]. However, here we will focus on the trauma care certification workshop of the summit that was presented by Indian, American, Israeli, and British academic clinicians.

On the first day of the Summit, Dr. Papadimos was the Shri. Tarsem Kurmar Garg Oration Inaugural Endowed Keynote speaker, and delivered a lecture entitled: *The Future of Predictive Modeling and its Contribution to Patient Safety*. Dr. Stawicki also presented a special lecture titled *Advanced Imaging in Post Mortem Setting: Is Autopsy Going Digital?*

Day one of the summit continued with a lecture series entitled the Adult ABC of resuscitation, although not required for the trauma and critical care certification course, it set the tone. The lectures discussed ACLS, stroke care guidelines, rapid sequence intubation and fluid and blood product resuscitation by Indian and US faculty. It served to introduce the attendees for the next 3- day Trauma and Critical Care Resuscitation Course (TCCRC) starting in the ED and continuing on to the operating room or the ICU.

The certification course started with the FORT (Fundamental of Resuscitation in Trauma) the afternoon of day one. The room was overflowing with medical students, nurses, and junior and senior level faculty from all disciplines to take part in this ATLS based trauma approach that was to be the foundation of the TCCRC.

Dr. Swaroop started the afternoon with a warm up case and discussion of the importance of collaboration which set the stage for the ABCs of Trauma.

Dr. Harry L. Anderson III, co-director of the Surgical Intensive Care unit and Surgical Critical Care Fellowship Program Director at St. Joseph Mercy Medical Center, Ann Arbor, Michigan, USA, then addressed the secondary survey and engaged the audience with interactive questions.

The course then focused on injuries by body system. Dr. Moshe Michaelson, Director Emeritus of the Trauma Unit and Department of EM at the Rambam Health Care Campus, Haifa, Israel started with the lecture on injuries specific to the head, intra and extradural, the face and the neck. Dr. Vineet Kumar, an Assistant Professor of Department of General Surgery at Lokmanya Tilak Municipal Medical College and General Hospital, Sion, Mumbai, India then continued with a lecture on chest trauma. Dr. Colin G. Kaide, an Associate Professor of EM from The Ohio State University, Columbus, Ohio, USA then gave a dynamic lecture on abdominal trauma intermingled with EM residency photographs. Dr. Stawicki then lectured about the extremes of age and the pregnant patient and the cautions that must be taken with each group.

The next speaker, Dr. Tamara Worlton, an Assistant Professor of Surgery at the Uniformed Services University of the Health Sciences, Bethesda, Maryland, USA, with active duty military experience as a member of a forward surgical team to care for critically wounded personnel in Afghanistan lectured about extremity blast and burn injuries that she has seen and that are common in many parts of India with domestic and international terrorism.

Dr. Himanshu Kataria, a Consultant in EM at St. Helens and Knowsley Teaching Hospitals NHS Trust and Dr. Ram Manohar, a Consultant in EM at Wirral University Hospitals NHS Trust in the North West region of the United Kingdom then led the skills session and the simulation. The audience was divided into 4 rotating groups. A trauma scenario simulation, pelvic binding, logrolling, and helmet removal were the skills performed at the stations. Trauma faculty manned each station, which allowed the audience one on one time and an opportunity to practice their comprehension of the didactics and ask questions.

Day two focused on resuscitation in the emergency room and more in-depth perspectives of critical care related to the EM patient. Dr. Kaide began the morning series with a presentation on trauma resuscitation. He presented landmark studies and evidenced-based guidelines, highlighting the clinical application. His lecture covered a variety of topics from airway management to massive transfusion in trauma.

Dr. Ravi S. Tripathi, an Assistant Professor of Anesthesiology and Critical Care from The Ohio State University, Columbus, Ohio, USA then presented a discussion of trauma specific to cardiac injury. His presentation on fundamentals of cardiac resuscitation focused on blunt cardiac injury as well as life threatening cardiac conditions such as cardiac rupture, fatal arrhythmias, and acute coronary syndrome leading to trauma.

In continuing with a hemodynamic focus, Dr. Anderson addressed managing patients with life-threatening infections, sepsis, and septic shock. Dr. Anderson highlighted key trials and publications that played heavily in the recent update of the Surviving Sepsis Campaign recommendations and their application to trauma patients.

The morning then changed focus to neurologic injury and trauma with a joint discussion by Dr. Stawicki and Dr. Tripathi. Dr. Stawicki laid the groundwork for the discussion in his presentation that focused on neurophysiologic principles and their practical application. The discussion of management of intracranial hypertension was especially useful. Dr. Tripathi followed Dr. Stawicki with a review of stroke care, both ischemic and hemorrhagic. The lecture focused on initial management of the stroke patients, stabilization, and establishing infrastructure for stroke care.

The morning concluded with a presentation by Dr. Michaelson. After the morning focused on individual organ injuries, Dr. Michaelson offered insight into patient triage, for both mass casualty as well as in a busy emergency department.

During the afternoon, workshops were hosted by both local medical school and visiting programs. These hand-on seminars focused on diagnostic modalities such as emergency and trauma ultrasound as well as therapeutic modalities integral to trauma care such as airway management, wound care, suturing, splinting, as well as toxicology life support.

### The last day of the course concentrated on the basics of bedside critical care

The critical care course has evolved significantly since its 2012 iteration at the 8th INDUS-EM Summit in Nashik, Maharasthra, India. Conceived by the OPUS 12 Foundation faculty, the course now includes international faculty from several high-profile academic institutions.

The 2013 course started with Dr. Anderson who addressed bedside and advanced diagnostics used to evaluate patients in the ICU. He was followed by Dr. Bahner who provided a perspective on the usage of ultrasonography at the bedside, with numerous video cases to highlight a plethora of abnormal findings. Dr. Kaide then presented a series of vignettes to emphasize disease processes that can be deadly if missed.

Dr. Swaroop continued the curriculum with a concise and comprehensive presentation on mechanical ventilatory support. There was a significant amount of discussion from the audience regarding the benefits and actual usage of rescue therapy in ARDS, focusing on capabilities of local hospitals to handle such complex patients.

Dr. Kapil Dev Soni, an Assistant Professor of Critical & Intensive Care at Jai Prakash Narayan Apex Trauma Centre, AIIMS, New Delhi then followed with a lecture about basic and advanced hemodynamic support. He discussed the theoretical and practical uses of various hemodynamic monitoring devices and vasoactive medications. This was followed by Dr. Worlton, who conducted a video presentation and a discussion of key techniques for performing bedside procedures, focusing on practical tips for safe and effective conduct of each respective procedure.

Dr. Stawicki ended the morning and concluded the course with a lecture on blast injury management, followed by a discussion of pitfalls and pearls in ICU care. The last session focused on common errors in trauma and critical care management, as well as on patient safety topics. The course ended with certificates given to the attendees of all three days of the course. Certificates of appreciation were also given to course faculty and directors.

### Conclusions and accomplishments

EM-Leader accomplishments included dissemination and instruction in techniques of scientific inquiry, including epidemiology and statistics and data acquisition; instruction on how to write grants and the administrative processes related to research and grant funding; the advantages of the electronic record and its potential uses in research; the ethical pitfalls in the scientific method, the funding of projects generally, and the protection of human subjects. In the trauma and critical care education portion, established life saving techniques in regard to monitoring, diagnosis, evaluation, and treatment were taught and this course was followed by a certification examination.

India has limited educational and clinical resources in regard to emergency medicine, trauma, and critical care. The medical educational value that was added to this population of students, residents, and young faculty included preparation for scientific inquiry and techniques of clinical intervention for successful care of the seriously ill patient. This curriculum will be presented through the next decade of INDUS-EM conferences. While the topic and themes of the Summit Program will change from year to year, the EM-leader preconference education and the trauma critical care portion will continue. As the conference changes its venue each year, a different portion of the country will be exposed to the above-mentioned (and valued) concepts.

Crucial next steps in the provision of appropriate emergency, trauma, and critical care in India will be the expansion of true emergency rooms that are staffed by competent practitioners in properly equipped facilities. “Casualty areas” must be replaced by state-of-the-art emergency facilities, and manned by appropriately trained physicians. For this to happen India and her friends such as the United Kingdom and the United States of America must assist in the training of Indian residents. In this vein, emergency medicine residents in India will now be admitted to the examination in Emergency Medicine by the appropriate medical educational organizations of the UK, and even permitted a minimal amount of training time in the UK. Thus, there will be training and some confirmation of minimal expertise as an expectation for those manning emergency facilities. These educational efforts must be persistent and consistent.

The next meeting will be the 10th anniversary of INDUS-EM and will be entitled, *Money, Management, and Manpower in Medicine*. It will be held in Lucknow, the capital city of Uttar Pradesh from October 15 to the 19th 2014. The focus will be the business of medicine with 15 tracks, 16 workshops and over 200 national and international faculty. A multifaceted topic that is sure to stimulate many controversial and enlightening conversations. The discussion of the business economics of medical practice in India, and the associated ethics, will involve a series of wide-ranging discourses that are currently in evolution. Such an intellectual intercourse will require the serious application of scientific, social, and ethical precepts to an evolving and growing technological marketplace in one the largest economies of the world.

The safe, effective, and ethical delivery of healthcare (in this case, emergency, trauma, and critical care) to all populations in India is what the Indian and international participants of INDUS-EM strive to promote and augment. Such an enterprise requires intense, repetitive, and inclusive efforts among all members of the medical and public health community, both in India and the greater global community.

## Competing interests

The authors declare that they have no competing interests.

## Authors’ contributions

All authors contributed equally. All authors read and approved the final manuscript.

## Authors’ information

All authors were lecturers at the conference as representatives of their particular institutions.
